# Effect of Heat Input on Interface Microstructure and Mechanical Properties of Al/Cu Laser Lap Welded Joints for Medium-Thickness Plates

**DOI:** 10.3390/ma19173627

**Published:** 2026-08-26

**Authors:** Peng Zeng, Wenzheng Dong, Qiong Li, Jie Yi, Xianghua Zhuo, Zheng Zeng

**Affiliations:** 1 School of Mechanical Engineering and Mechanics, Xiangtan University, Xiangtan 411105, China; 13627316886@163.com (P.Z.); zhuoxh2022@163.com (X.Z.); m18890191129@163.com (Z.Z.); 2 School of Mechanical Engineering, Hunan Industry Polytechnic, Changsha 410082, China; yifeng8457@163.com (Q.L.); 15111270111@163.com (J.Y.); 3 Hunan Provincial Engineering Research Center for Digital Technology of NC Machining Process, Changsha 410082, China

**Keywords:** laser welding, Al/Cu joint, intermetallic compound, interface microstructure, joint performance

## Abstract

To meet the demands for lightweight design and high-conductivity connections in new energy vehicles, the high-quality joining of dissimilar Al/Cu metals has emerged as a critical research focus. In this study, laser welding was performed on 2 mm-thick 1060 pure aluminum and T2 copper plates. The effects of laser power (3.6–4.0 kW) and welding speed (0.9–1.5 m/min) on the interfacial microstructural evolution and mechanical properties of the lap joints were systematically investigated. The results demonstrate that the macroscopic morphology of the weld is primarily governed by heat input: excessive laser power induces transverse cracking, whereas an overly low welding speed promotes porosity. Microstructural analysis revealed that intermetallic compounds (IMCs), such as Al_2_Cu, AlCu, and Al_4_Cu_9_, predominantly form at the interface, with their morphology and distribution varying significantly depending on the heat input. Under the optimized parameters of a 3.8 kW laser power and a 1.2 m/min welding speed, sufficient mixing of the molten Al and Cu was achieved. This promoted the formation of fine, dispersed IMCs accompanied by a continuous Al–Cu eutectic layer at the interface, yielding a maximum tensile-shear load of 1561 N. This research elucidates the intrinsic relationship between heat input and the microstructure–property correlation of Al/Cu laser-welded joints, identifying a viable process window for 2 mm-thick sheets and providing theoretical and practical guidance for joining dissimilar medium-thickness metal plates.

## 1. Introduction

Copper is extensively utilized in power electronics, energy and chemical engineering, and national defense due to its exceptional electrical and thermal conductivity, ductility, and corrosion resistance, making it a highly consumed critical metallic material [1,2]. However, copper is a costly and relatively scarce resource; thus, reducing its utilization cost is imperative in modern manufacturing [3]. Aluminum, which is abundant, cost-effective, and highly ductile with favorable electrical conductivity, serves as an ideal substitute [4,5]. The drive for lightweight construction and high-conductivity connections, particularly in emerging sectors like new energy vehicles, has accelerated the technological trend of substituting copper with aluminum. Consequently, Al/Cu dissimilar structures offer significant engineering value by balancing performance with cost efficiency [6,7,8].

Commercially available Al and Cu metals cover a wide range of alloy systems: aluminum alloys include 1xxx series pure aluminum and 6xxx series wrought alloys with varying Si, Mg, and Cu contents, while copper materials include T2 pure copper, brass and bronze, whose compositional differences significantly affect weldability and intermetallic compound (IMC) evolution. In industrial applications, Al and Cu components are often coated with oxide films, Ni, Ag, or Cu platings for corrosion protection and conductivity improvement, which further complicate the welding interface. In addition, recycled Al and Cu components containing impurity elements such as Fe, Si, and Zn are increasingly used in production; these impurities can alter molten pool fluidity and promote the formation of brittle IMCs, which is a non-negligible issue in engineering practice. In this study, uncoated AA1060 pure aluminum and T2 pure copper were selected as base materials to eliminate the interference of alloying elements, coatings, and impurities, so as to reveal the intrinsic regulation mechanism of heat input on interfacial metallurgical reactions and provide a benchmark for subsequent research on coated or recycled materials.

Various joining technologies have been developed for Al/Cu dissimilar metals, each with distinct advantages and limitations. Friction stir welding (FSW), as a solid-state joining method, can effectively suppress the formation of brittle IMCs, but it suffers from high clamping requirements, poor adaptability to complex structures, and low production efficiency. Fusion-brazing processes such as TIG and plasma arc welding have high process maturity, but their low energy concentration leads to large workpiece deformation and limited joint strength. In contrast, laser welding is recognized as a promising route for high-efficiency and high-quality joining of medium-thickness Al/Cu plates due to its high energy density, precisely controllable heat input, and high automation level.

The primary challenge in joining dissimilar Al/Cu metals stems from their substantial differences in melting point, thermal conductivity, and metallurgical compatibility. Conventional fusion welding, which melts both base metals, typically generates a massive amount of brittle Al-Cu intermetallic compounds (IMCs) within the weld seam, severely degrading the mechanical properties of the joint. Conversely, laser welding—characterized by precisely controllable heat input, high energy density, and a highly localized heating zone—is an effective method for achieving high-quality Al/Cu joints [9,10]. With a properly modulated heat input, the lower-melting-point aluminum can be fully melted while controlling the penetration depth on the copper side, thereby yielding a tailored interfacial microstructure.

Considerable progress has been made recently in the laser welding of Al/Cu dissimilar metals. For instance, Solchenbach and Plapper [11] demonstrated the feasibility of fiber laser welding-brazing on 500 µm-thick SF-Cu and EN-AW1050A aluminum sheets in an Al-on-Cu configuration. Weigl et al. [12] joined Al and Cu by incorporating AlSi12 and CuSi3 interlayers, showing that the maximum bending deformation of the joint nearly tripled the reference value, with a concurrent reduction in fracture surface roughness. Salimi et al. [13] employed ultrasonic-enhanced laser welding to redistribute IMCs, thereby enhancing the mechanical properties of welds for electric vehicle battery packs. Furthermore, Peng et al. [14] achieved Al/Cu plasma arc welding-brazing using a Zn-Al filler metal, attaining a joint shear strength of 175.5 MPa; their work highlighted significant elemental diffusion between the filler and base metals, with fractures localizing at the filler/Cu interface.

Existing studies on Al/Cu laser welding can be systematically divided into three technical routes. The first is filler-free laser welding, which features the simplest process and lowest cost, but its joint performance is restricted by the excessive growth of brittle IMCs [11,15]. The second is laser welding with filler wires or interlayers, which can significantly improve joint strength and ductility by regulating interfacial composition, but inevitably increases process complexity and material cost [12,14]. The third is energy field-assisted laser welding represented by ultrasonic vibration, which refines IMCs and enhances toughness by intensifying molten pool convection, yet requires additional equipment modification and is difficult to adapt to large-scale industrial production [13,16].

Therefore, achieving high-performance Al/Cu laser-welded joints solely through heat-input regulation, without filler metals or intermediate coatings, is of significant practical importance for reducing industrial production costs and simplifying the process chain, provided that joint performance is adequately maintained.

Further literature statistics indicate that most existing studies focus on thin sheets with a thickness of ≤1 mm, targeting scenarios such as microelectronic packaging and battery tabs [11,13,15]. For 1.5–3 mm medium-thickness plates widely used in new energy busbars and power connectors, systematic research on the process–microstructure–performance correlation of laser welding is still very limited. The IMC evolution law and its precise regulation mechanism under medium-thickness plate conditions have not been clearly clarified, which constitutes the core research gap addressed by this work.

In addition to filler and interlayer methods, thin intermediate coatings such as Ni, Ag and Cu plating are also widely used to regulate interfacial IMCs by blocking direct Al-Cu contact. However, these coating methods inevitably increase the preparation processes and material costs, and the coating-base metal interface may become a new failure risk point. Therefore, achieving high-performance Al/Cu joints only through heat-input regulation without fillers or intermediate coatings has important practical value for low-cost automated mass production.

To address this, the present study utilized 2 mm-thick AA1060 aluminum and T2 copper to systematically investigate how laser power and welding speed dictate the type, morphology, and distribution of interfacial IMCs. The objective is to achieve controllable optimization of the interfacial microstructure via heat-input regulation, supplying a theoretical foundation for the laser welding process design of medium-thick Al/Cu plates.

## 2. Materials and Methods

The base materials selected for this study were AA1060 aluminum and T2 pure copper, supplied as plates with dimensions of 200 mm × 100 mm × 2 mm in a fully annealed (O-temper) condition. Their chemical compositions are detailed in Table 1 and Table 2, respectively. Prior to welding, the plate surfaces were ground with 500-grit SiC abrasive paper to remove oxide films and subsequently wiped with acetone to eliminate residual contaminants. The AA1060 pure aluminum exhibits a tensile strength of 76 MPa with an elongation of 27%, while the T2 pure copper exhibits a tensile strength of 242 MPa with an elongation of 8%.

**Figure 1:**
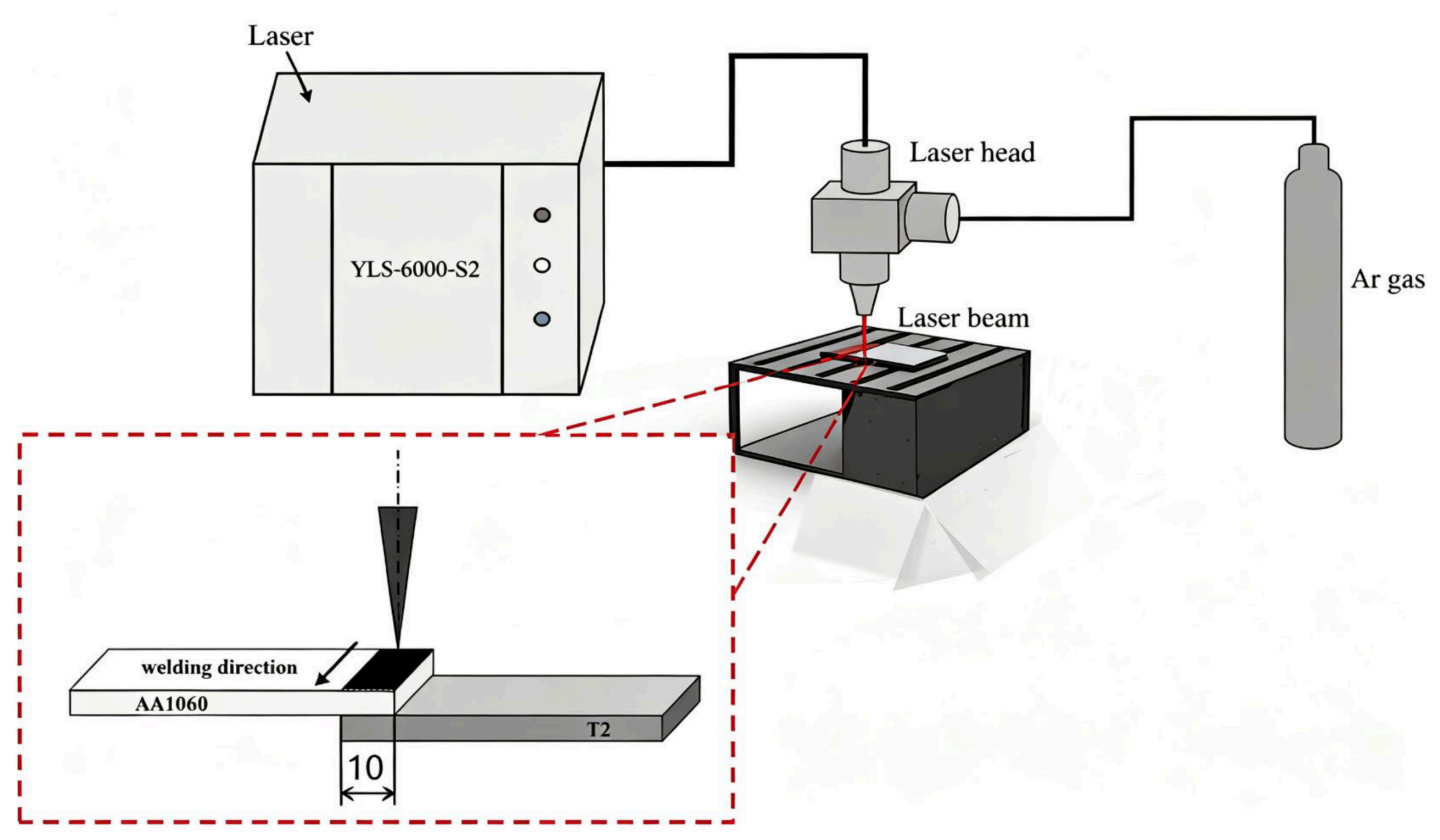
Schematic diagram of welding.

Laser welding of AA1060 aluminum and T2 copper plates was carried out in a lap configuration with aluminum on top and copper on the bottom. The lap width was 10 mm, and a schematic diagram of the welding setup is shown in Figure 1. The laser welding equipment used was an IPG YLS-6000-S2-TR fiber laser (IPG Photonics Corporation, Marlborough, MA, USA) with a maximum output power of 6 kW and a wavelength of 1070 nm. The focusing lens had a focal length of 300 mm, and the focused spot diameter was 0.3 mm, corresponding to a power density of approximately 5.4 × 10^6^ W/cm^2^ under the optimal process parameter. Welding was performed using an ABB robot. At the start of welding, the laser beam was focused onto the AA1060 aluminum plate with a deflection angle of 10° from the vertical direction to prevent back-reflection. The process parameters were designed based on the principle of ensuring complete melting of the AA1060 aluminum side to form a molten pool, while controlling the heat input to induce only partial melting of the T2 copper side without full penetration of the copper base metal, thereby achieving metallurgical bonding between Al and Cu. The laser power ranged from 3.6 to 4.0 kW, and the welding speed ranged from 0.9 to 1.5 m/min. The defocusing distance was set to zero. During welding, argon gas was used to provide full protection for the molten pool, the back side of the weld, and the gradually cooling weld metal, with a gas flow rate of 25 L/min on both sides. The detailed welding parameters and corresponding sample designations are listed in Table 3. To quantitatively describe the effect of heat input on weld formation and microstructure, the heat input E was defined as E = 60 P/v, where P is the laser power (kW), v is the welding speed (m/min), and the unit of E is kJ/m.

Post-welding, metallographic cross-sections were cut perpendicular to the welding direction and mounted in acrylic resin. The specimens were sequentially ground using SiC papers (up to 3000 grit) and polished with a 0.5 μm diamond suspension. The polished surfaces were then etched with Keller’s reagent (2.5 mL HNO_3_ + 1.5 mL HCl + 1.0 mL HF + 95 mL H_2_O). Phase analysis was conducted using X-ray diffraction (XRD) operating at 40 kV and 40 mA, with a scanning rate of 3°/min over a 20–100° range. A Cu Kα radiation source with a wavelength of 0.15406 nm was adopted. The microstructural and morphological characteristics were observed via scanning electron microscopy (SEM, FEI Quanta 200, FEI Company, Hillsboro, OR, USA) equipped with backscattered electron (BSE) imaging and energy-dispersive X-ray spectroscopy (EDS, Oxford AZtec 3.0 software). EDS point analysis was performed at an accelerating voltage of 20 kV and a working distance of 10 mm, with a spot size of 3 μm. Pure Al and pure Cu standard samples were used for quantitative calibration before each test. Cross-sectional dimensions, elemental mixing behavior, and interfacial IMC distribution were quantitatively evaluated using image analysis software (Oxford AZtec 3.0 software).

To evaluate the shear performance of the joints, rectangular tensile specimens with a width of 30 mm were machined perpendicular to the welding direction using wire electrical discharge machining (WEDM). The macroscopic lap width was set to 10 mm, but only the laser-welded fusion zone formed effective metallurgical bonding at the Al/Cu interface. Therefore, the nominal shear strength τ was calculated based on the effective bonding area, using the following equation: τ = F_max_/(w_weld_ × B), where F_max_ is the peak tensile-shear load, w_weld_ is the effective weld width at the Al/Cu interface measured from typical metallographic cross-sections, and B is the specimen width (30 mm). Three replicate specimens were tested for each parameter set. Tensile tests were executed on a universal testing machine at a crosshead speed of 1 mm/min. All tests were carried out under standard laboratory conditions with a temperature of 25 ± 2 °C and a relative humidity of 45–55%. Results are reported as “mean ± standard deviation (SD)”, with error bars illustrating data dispersion. Following the tests, the fracture surfaces on both the aluminum and copper sides were analyzed using SEM to identify the fracture modes and trace the crack propagation paths relative to the IMC distribution.

## 3. Results

### 3.1. Weld Macromorphology

**Figure 2:**
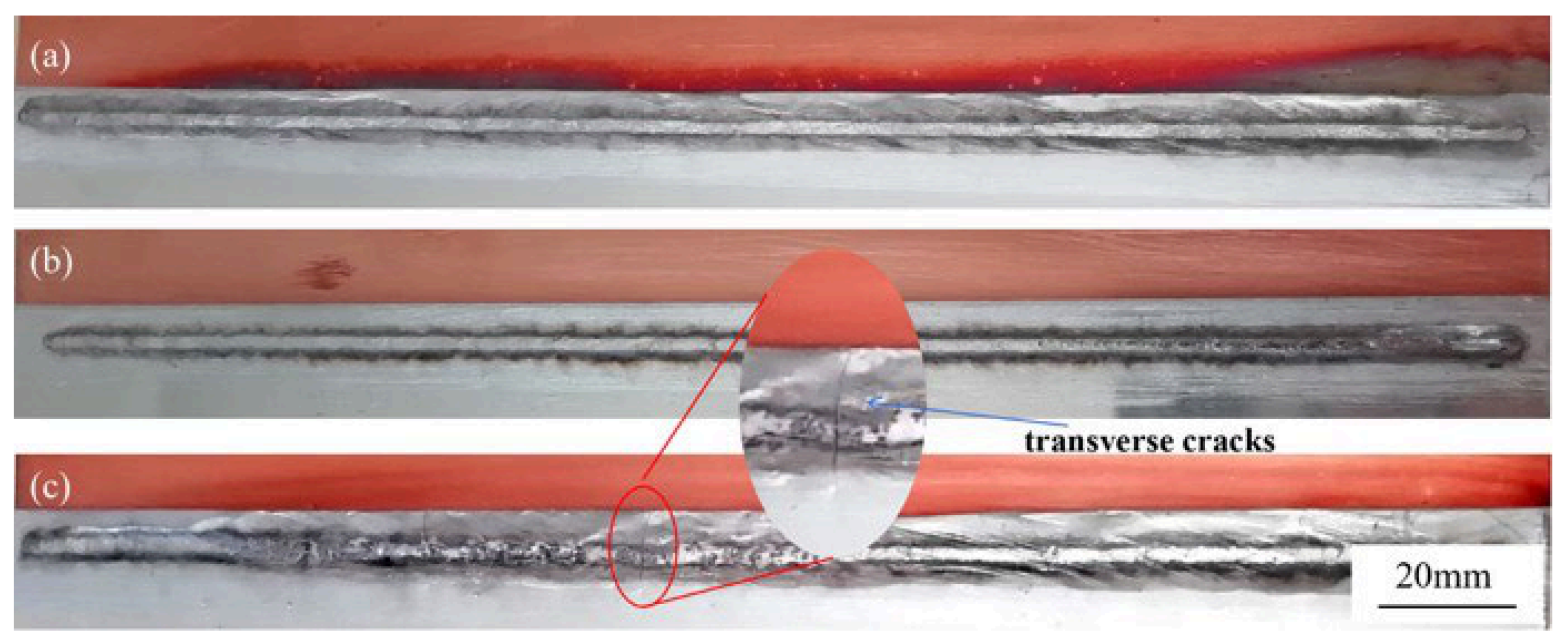
Macromorphologies of the welded joints at different laser powers with a welding speed of 1.2 m/min: (**a**) weld surface of No. P1S2 at 3.6 kW; (**b**) weld surface of No. P2S2 at 3.8 kW; (**c**) weld surface of No. P3S2 at 4.0 kW.

Figure 2a–c displays the surface morphologies of the Al/Cu welds fabricated at a constant welding speed of 1.2 m/min with varying laser powers (3.6, 3.8, and 4.0 kW). At 3.6 kW, the weld surface was continuous and relatively flat, exhibiting a smooth finish without apparent defects. At 3.8 kW, the surface uniformity slightly decreased, though structural continuity was preserved. However, at 4.0 kW, the weld surface became irregular, and distinct transverse cracks emerged. This indicates that excessive laser power alters the heat input and solidification dynamics, intensifying internal stress concentration on the aluminum side and ultimately inducing transverse cracking due to the severe temperature gradient and rapid solidification.

**Figure 3:**
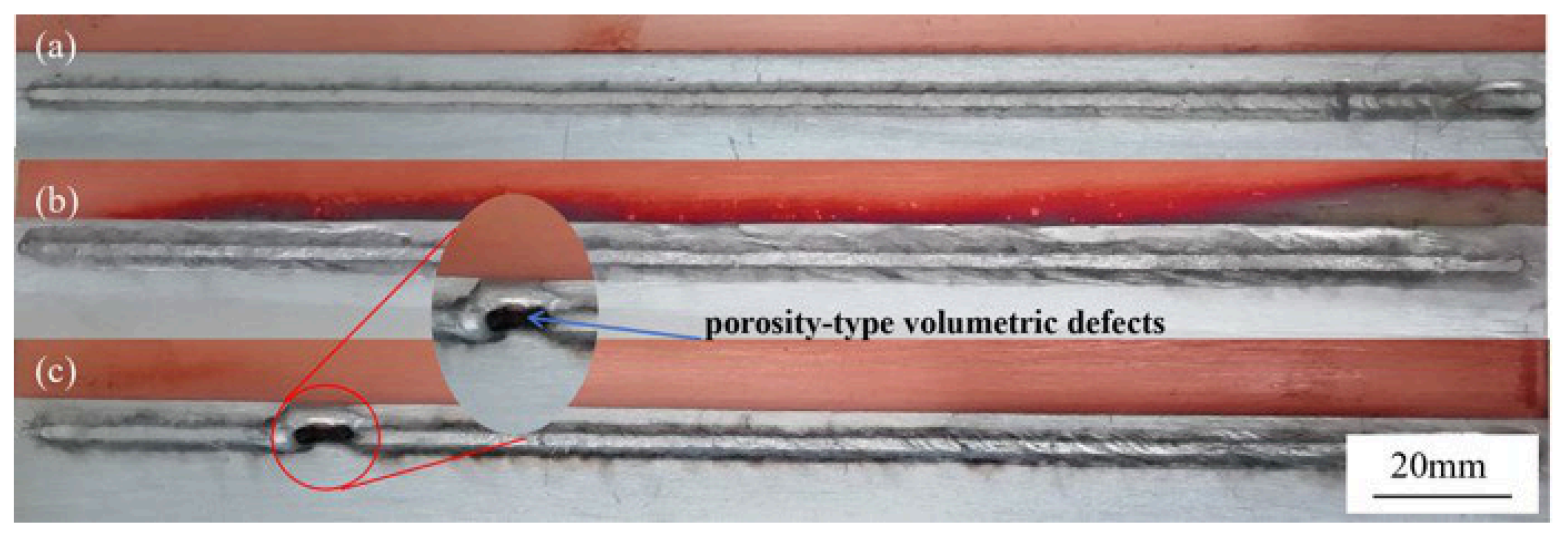
Macromorphologies of the welded joints at different welding speeds with a laser power of 3.6 kW: (**a**) weld surface of No. P1S1 at welding speed 1.5 m/min; (**b**) weld surface of No. P1S2 at welding speed 1.2 m/min; (**c**) weld surface of No. P1S3 at welding speed 0.9 m/min.

Figure 3a–c presents the surface morphologies at a constant laser power of 3.6 kW across different welding speeds (1.5, 1.2, and 0.9 m/min). At 1.5 m/min, the weld was continuous and uniform, free of surface or volumetric defects. Reducing the speed to 1.2 m/min marginally increased the surface roughness without compromising integrity. As the welding speed decreased, the heat input increased, causing a significant rise in molten pool temperature and a longer molten pool lifetime. Since the melting point (660 °C) and boiling point (2467 °C) of aluminum are much lower than those of copper (melting point 1084 °C), excessive molten pool temperatures can lead to local overheating or even vaporization of the aluminum liquid. The resulting gas phase struggles to escape completely before solidification of the molten pool. Additionally, the prolonged high temperature enhances melt flowability and intensifies fluctuations, easily inducing metallurgical inhomogeneity within the aluminum liquid, and ultimately leading to the formation of volumetric defects in the weld.

**Figure 4:**
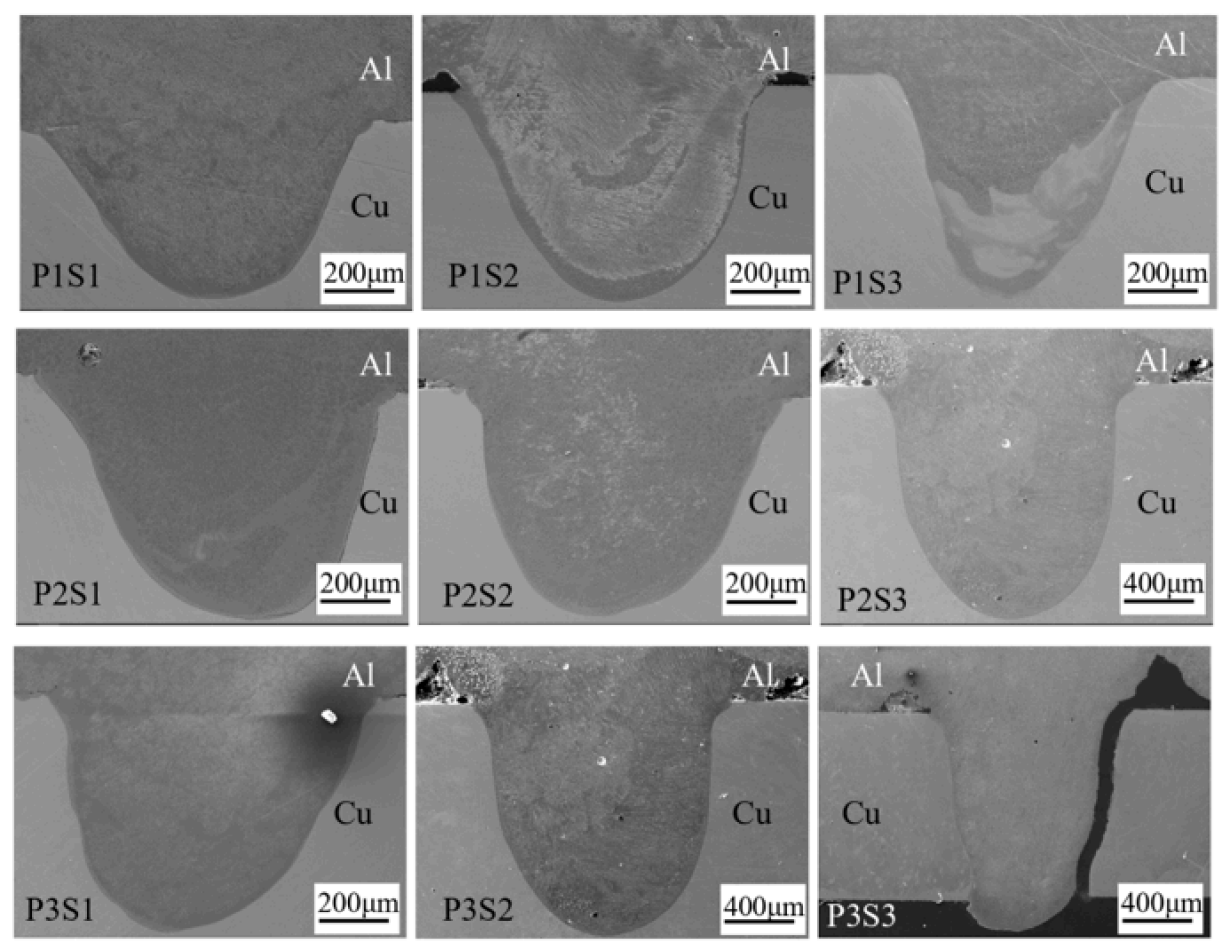
Secondary electron (SE) image of the weld cross-section.

**Figure 5:**
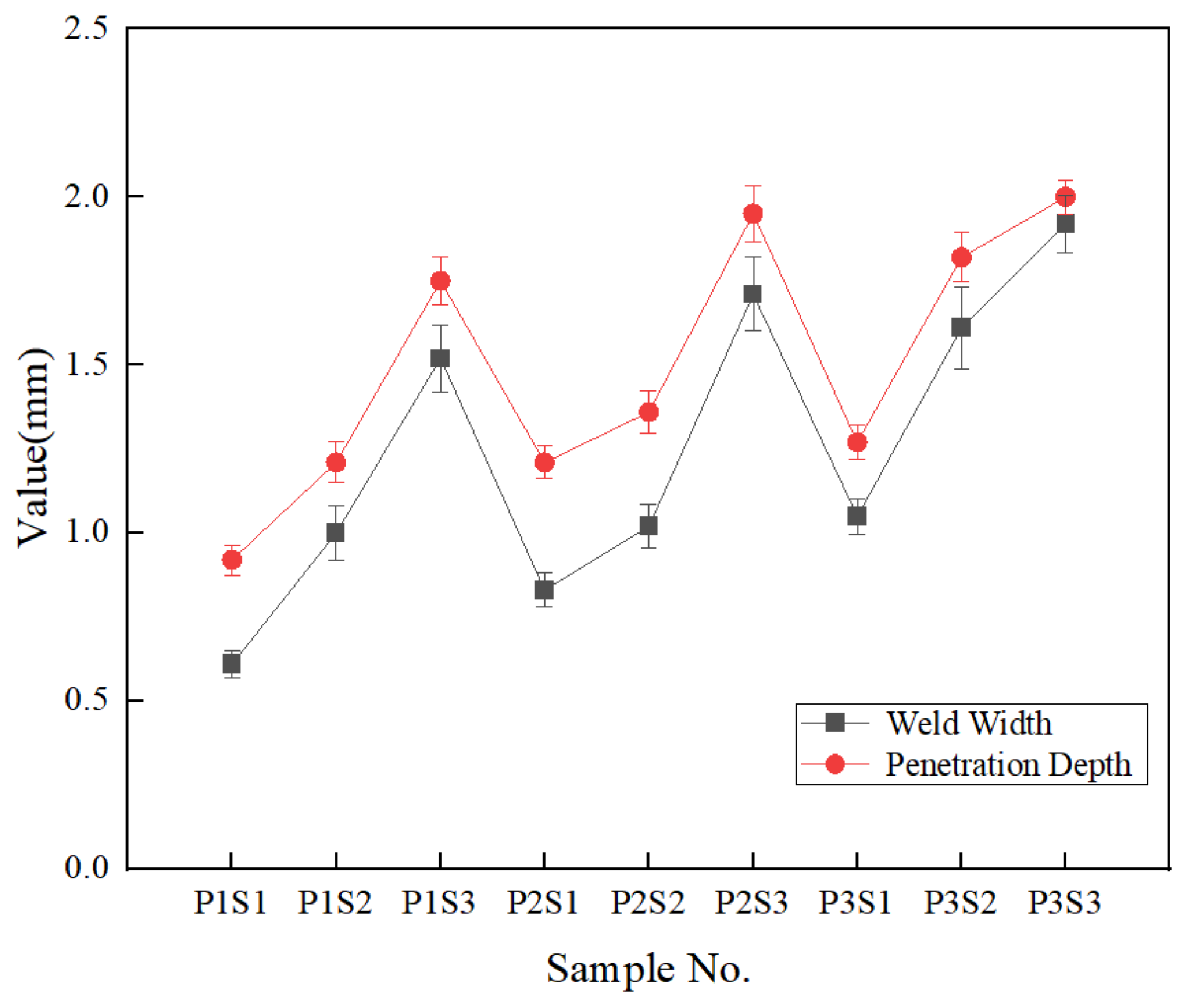
Statistical results of weld width and penetration depth (error bars represent standard deviation, *n* = 3). Sample nomenclature: P stands for laser power level, S stands for welding speed level; e.g., P1S1 corresponds to 3.6 kW + 1.5 m/min.

Figure 4 shows the secondary electron (SE) images of the weld cross-section, where the boundaries of the molten pool and the weld seam can be clearly observed. Statistical analyses were performed on the weld width and penetration depth of the weld cross-section, and the results were obtained by averaging the measured values from three specimens, as shown in Figure 5. In the 3.6 kW power group (P1 series), as the welding speed decreased from 1.5 m/min (P1S1) to 0.9 m/min (P1S3), the penetration depth on the aluminum side increased from approximately 0.9 mm to 1.7 mm, and the weld width increased from approximately 0.6 mm to 1.5 mm. For the 3.8 kW power group (P2 series), the P2S2 sample exhibited an aluminum-side penetration depth of approximately 1.3 mm and a weld width of approximately 1.0 mm; at the lowest welding speed (P2S3), the penetration depth reached about 1.9 mm with a weld width of 1.7 mm, with further deepened melting on the copper side. In the 4.0 kW power group (P3 series), the P3S3 sample had the largest values among all groups, with a penetration depth of approximately 2.0 mm (full penetration on the aluminum side) and a weld width of approximately 1.9 mm, accompanied by excessive local melting on the copper side.

This evolution can be attributed to the coupled thermal effect of laser power and welding speed. Increasing the laser power or decreasing the welding speed both elevate the heat input, enhancing the melting of the aluminum base metal and intensifying the convective mixing of the Al and Cu molten pools, thereby enlarging the molten pool size on the aluminum side and the degree of melting on the copper side. However, excessive heat input (e.g., 4.0 kW + 0.9 m/min for P3S3) induces superheating of the molten pool, which not only aggravates aluminum vaporization and gas phase entrapment (leading to porosity), but also results in an excessively thick interfacial IMC layer and solidification stress concentration, ultimately deteriorating the cross-sectional quality of the joint.

### 3.2. Interfacial Microstructure and Composition Analysis

**Figure 6:**
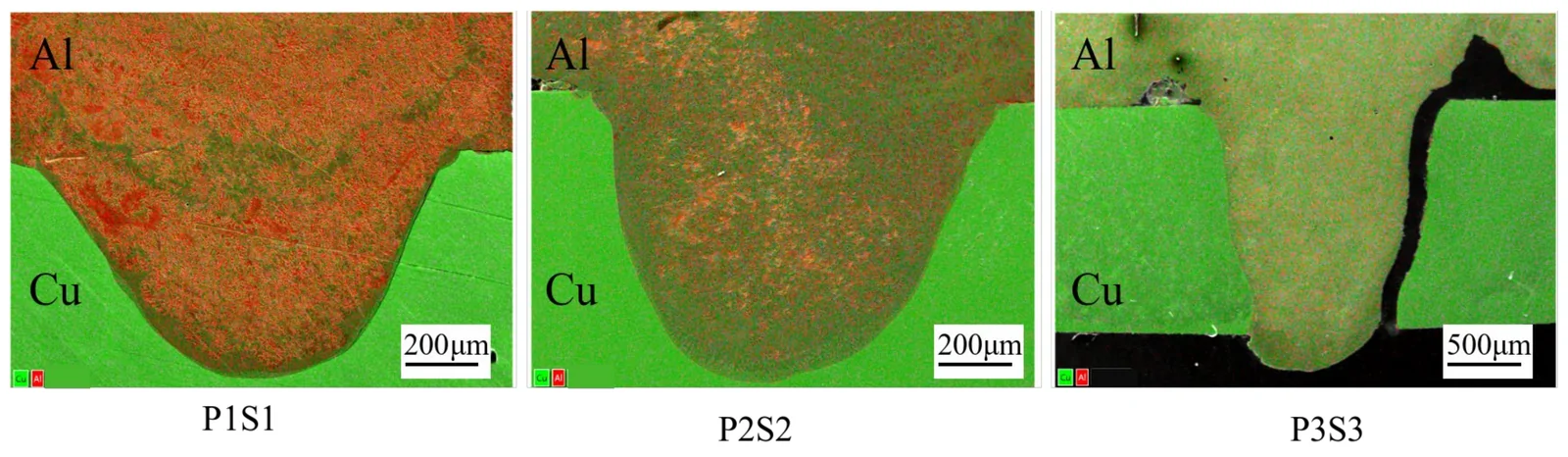
Element distribution maps of Al and Cu in joint cross-sections under three typical parameters.

Three typical parameter combinations, namely P1S1 (3.6 kW—1.5 m/min, low heat input), P2S2 (3.8 kW—1.2 m/min, moderate heat input with optimal performance), and P3S3 (4.0 kW—0.9 m/min, excessive heat input), were selected for detailed SEM/EDS microstructural analysis. These three samples uniformly cover the full range of heat input from low to high, and can fully reflect the evolution law of interfacial IMCs with heat input. The other six parameter groups were used as auxiliary verification samples, and their microstructural evolution trends are consistent with the three typical samples. The Al-Cu mixed structure and the distribution of intermetallic compounds (IMCs) at the interface were characterized using scanning electron microscopy and energy dispersive X-ray spectroscopy (SEM-EDS). Figure 6 shows the elemental mapping results of the joints, where red and green represent Al and Cu, respectively. Al-Cu mixing mainly occurs in three regions of the weld cross-section: the upper Al layer, the middle Al-Cu interface, and the lower Cu layer, with the most intense mixing observed at the Al-Cu interface. The degree of mixing primarily depends on the melting amount of the base metals on both sides and the flow behavior of the molten pool, which in turn determine the distribution of IMCs at the Al–Cu interface. Therefore, at a low welding heat input (e.g., the P1S1 joint), the melting amount of the copper side is insufficient, resulting in the incomplete mixing of Al and Cu. In contrast, for joints with a larger penetration depth (e.g., the P3S3 joint), the melting amount of the copper side increases, and the Al-Cu mixed region expands accordingly.

**Figure 7:**
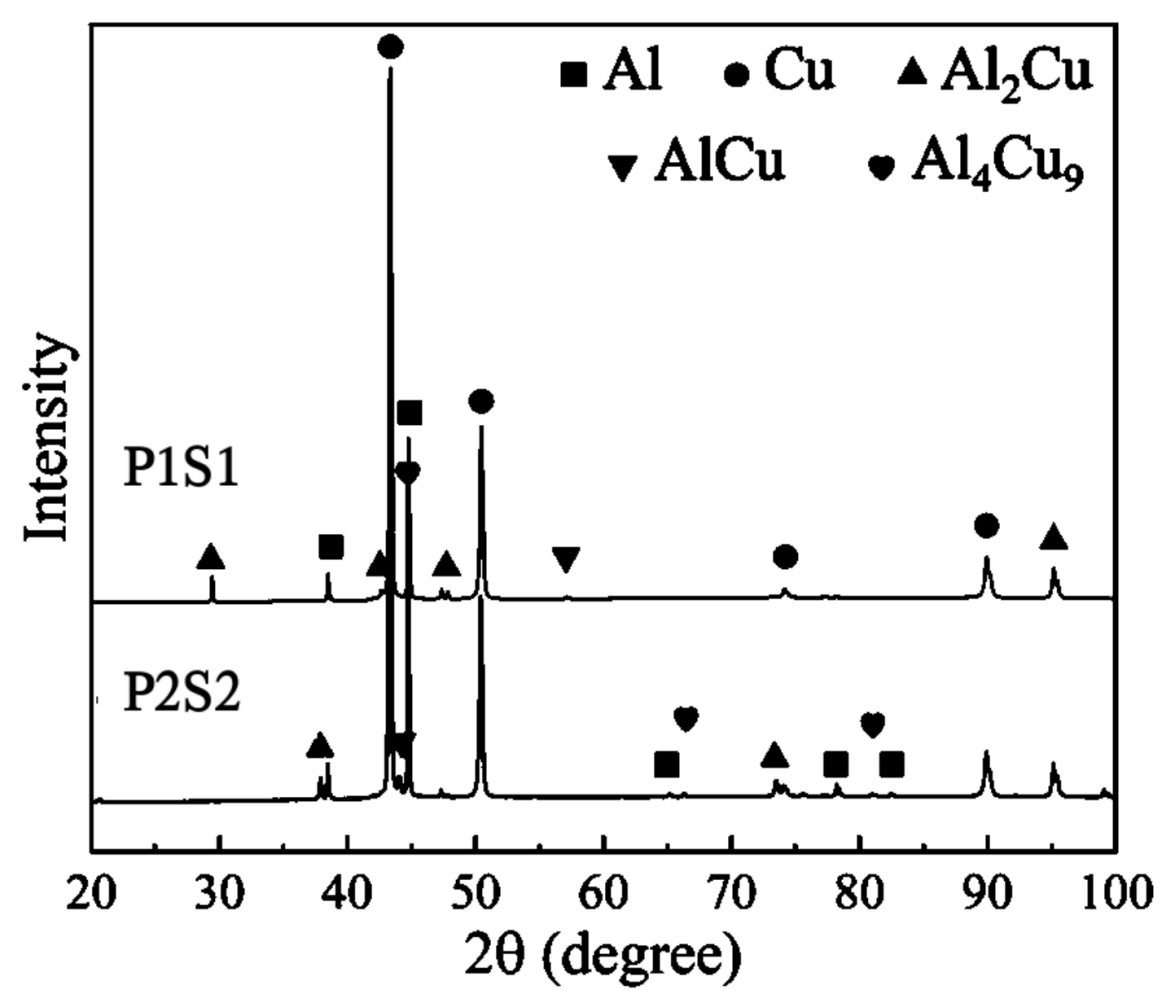
X-ray diffraction (XRD) patterns.

In the weld seam, the mixture of Al and Cu eventually forms various intermetallic compounds (IMCs). Figure 7 shows the X-ray diffraction (XRD) patterns obtained from the cross-sections of the P1S1 and P2S2 welds. In addition to the diffraction peaks of the base materials, Al_2_Cu, AlCu, and Al_4_Cu_9_ phases were detected in the joints, with Al_2_Cu exhibiting the strongest diffraction peak, indicating that this phase is readily formed in the joint.

**Figure 8:**
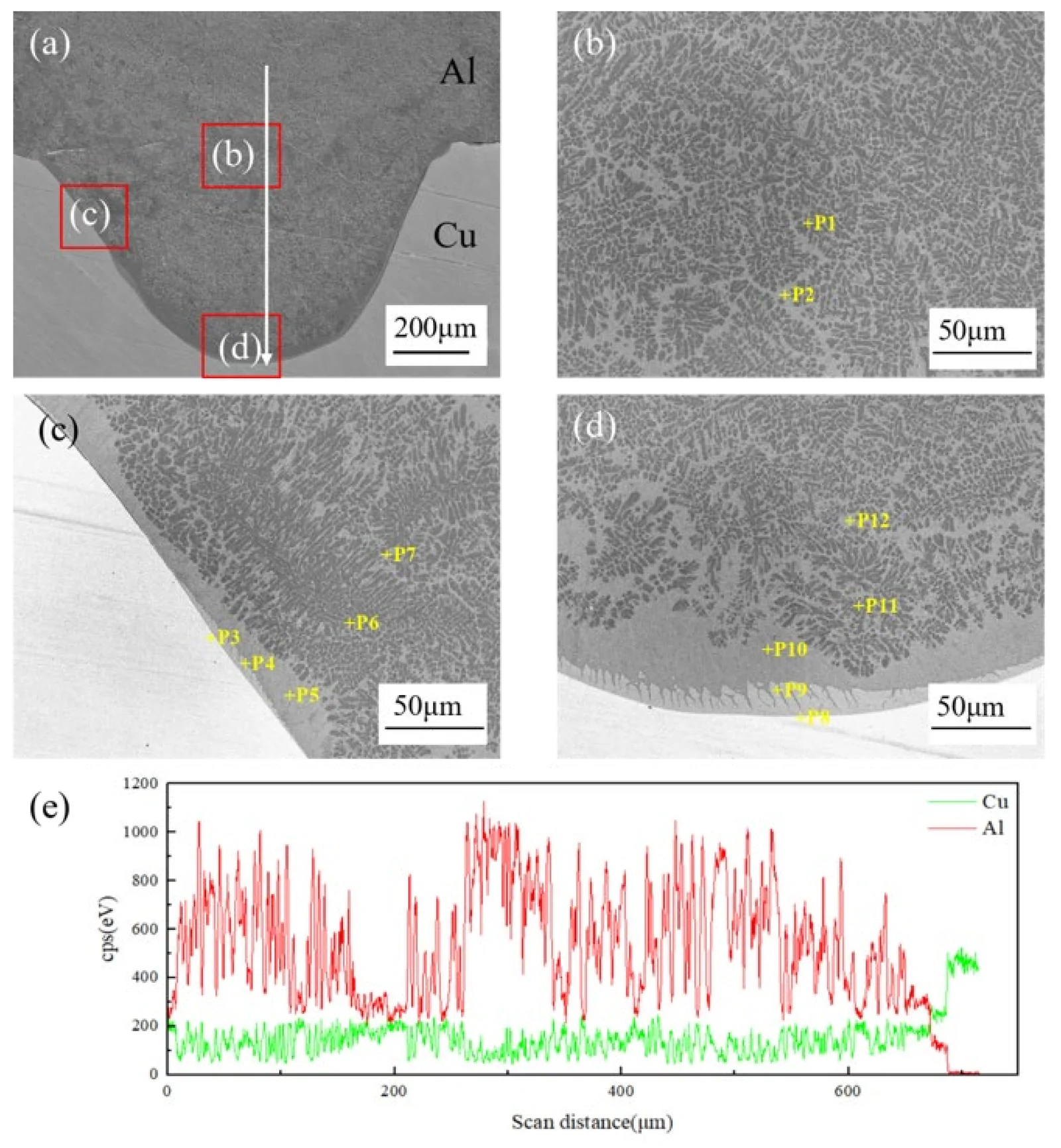
SEM/EDS analysis of the joint at parameters of 3.6 kW laser power + 1.5 m/min welding speed: (**a**) macroscopic SEM image of the joint; (**b**) magnified view of the middle region in (**a**); (**c**) magnified view of the shoulder interface region in (**a**); (**d**) magnified view of the bottom interface region in (**a**); (**e**) EDS line scan result of the weld, the position and direction of the line scan are shown in (**a**), along with the lines and arrows. The yellow “+” marks represent EDS test points, numbered 1–12, whose chemical compositions and phase identifications correspond to Table 4.

**Figure 9:**
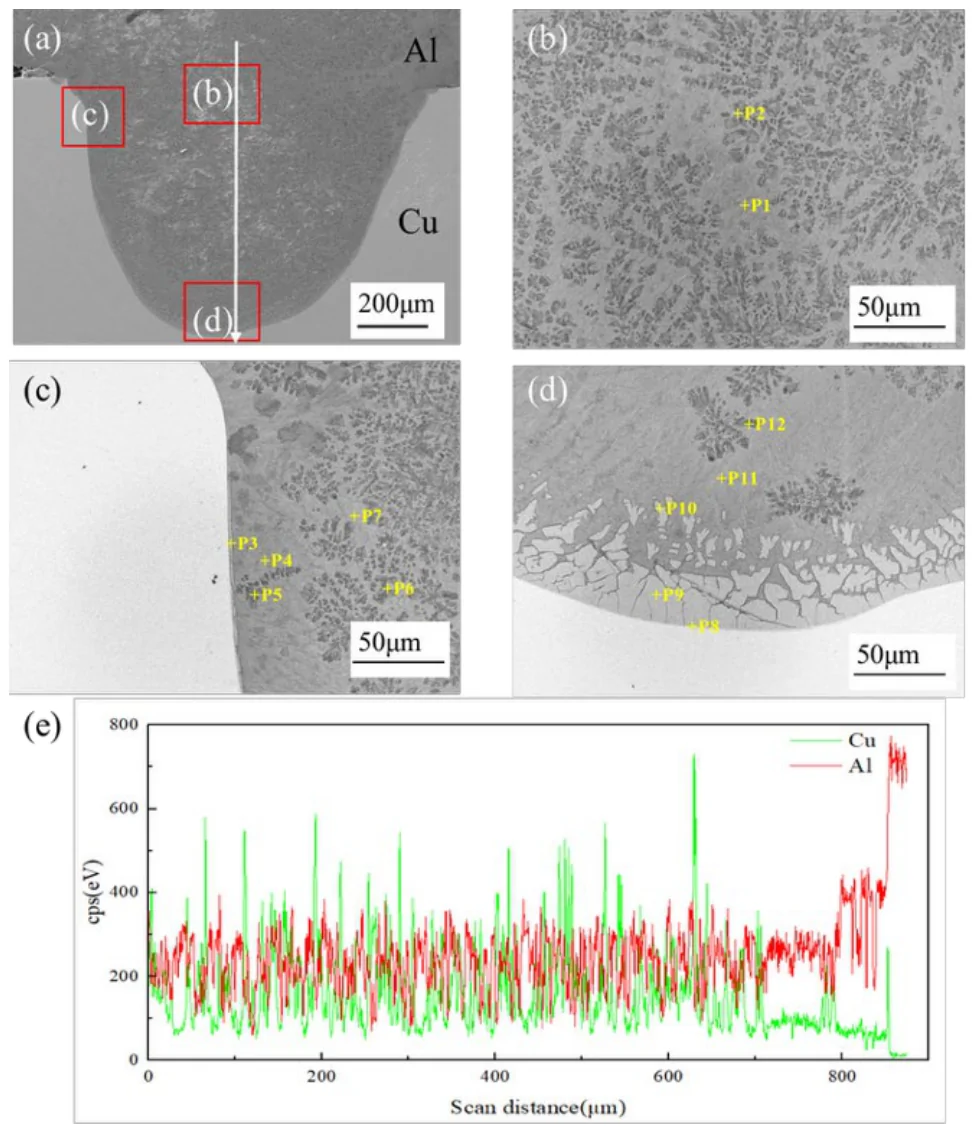
SEM/EDS analysis of the joint at parameters of 3.8 kW laser power + 1.2 m/min welding speed: (**a**) macroscopic SEM image of the joint; (**b**) magnified view of the middle region in (**a**); (**c**) magnified view of the shoulder interface region in (**a**); (**d**) magnified view of the bottom interface region in (**a**); (**e**) EDS line scan result, the position and direction of the line scan are shown in (**a**), along with the lines and arrows. The yellow “+” marks represent EDS test points, numbered 1–12, whose chemical compositions and phase identifications correspond to Table 5.

**Figure 10:**
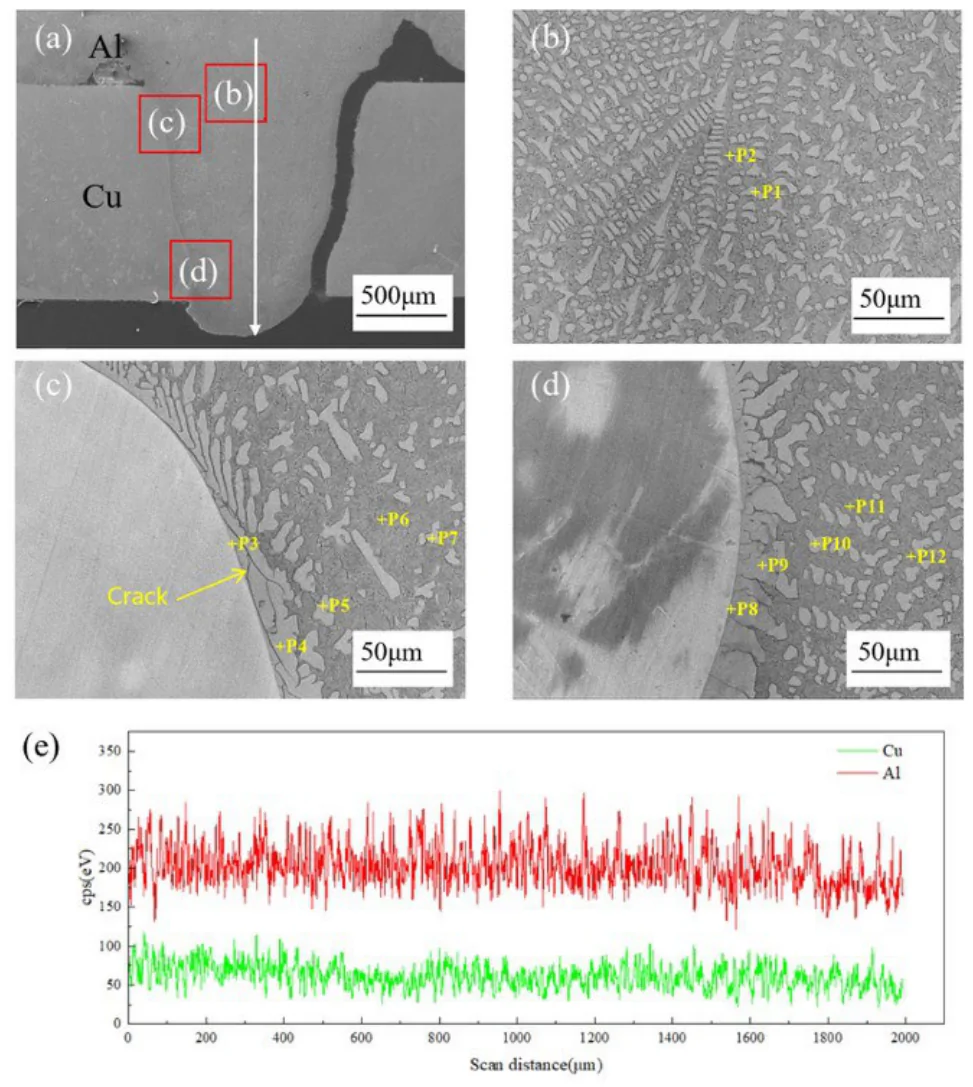
SEM/EDS analysis of the joint at parameters of 4.0 kW laser power + 0.9 m/min welding speed: (**a**) macroscopic SEM image of the joint; (**b**) magnified view of the middle region in (**a**); (**c**) magnified view of the shoulder interface region in (**a**); (**d**) magnified view of the bottom interface region in (**a**); (**e**) EDS line scan result, the position and direction of the line scan are shown in (**a**), along with the lines and arrows. The yellow “+” marks represent EDS test points, numbered 1–12, whose chemical compositions and phase identifications correspond to Table 6.

Since aluminum melting occurred in all specimens, the degree of melting on the copper side, the mixing behavior of the Al/Cu molten pool, and the distribution characteristics of interfacial IMCs are closely correlated. To further clarify the interfacial microstructure, particularly the characteristics of IMCs, high-magnification backscattered electron (BSE) images of the interfacial region under three typical parameter combinations (3.6 kW—1.5 m/min, 3.8 kW—1.2 m/min, and 4.0 kW—0.9 m/min) were obtained using SEM/EDS, as shown in Figure 8, Figure 9 and Figure 10, to investigate the effects of different laser powers and welding speeds on the interfacial microstructural evolution.

As shown in Figure 8, the weld microstructure obtained under the processing parameters of laser power 3.6 kW and welding speed 1.5 m/min is presented, where Figure 8a shows the macromorphology of the weld. EDS line scanning was performed along the top-to-bottom direction at the weld center (Figure 8e), and EDS point analyses were conducted at the middle weld region (Figure 8b), the shoulder interfacial region (Figure 8c), and the bottom interfacial region (Figure 8d). The chemical compositions at the yellow “+” marked points in each region are listed in Table 4. Due to the sensitivity of backscattered electron (BSE) contrast to atomic number, the bright white regions correspond to Cu-rich phases, the dark gray regions correspond to Al-rich phases, and the intermediate gray regions correspond to Al-Cu intermetallic compound (IMC) layers. Moreover, noticeable differences in microstructure can be observed across different regions of the weld.

As shown in Figure 8b, the middle region of the weld was mainly composed of black dendritic structures and graynet structures. Point 1, located in the black dendritic structure, had a Cu content of only 4.61 at.%. Based on the Al-Cu binary phase diagram, this phase can be identified as α-Al. Point 2, located in the gray network-like structure, had a Cu content of 35.42 at.%, corresponding to a mixed phase of α-Al + Al_2_Cu.

The shoulder interfacial region near the copper side, shown in Figure 8c, exhibited four types of phases with different contrasts and morphologies. Point 3, located in the transition zone, had a Cu content as high as 76.17 at.%, corresponding to a mixed phase of Cu + Al_4_Cu_9_. Point 4 was tentatively identified as a narrow strip-like structure with a chemical composition matching the AlCu phase. Point 5 was tentatively identified as a thicker gray structure, identified as the Al_2_Cu phase. Points 6 and 7 corresponded to structures similar to the main weld body, where Point 6 was the α-Al + Al-Cu eutectic phase, and Point 7 was a mixed phase of α-Al + Al_2_Cu. In this region, numerous columnar, continuously distributed Al_2_Cu IMCs perpendicular to the welding direction could also be observed. Additionally, strip-like Al_2_Cu IMCs extending along the weld were present at the interface between the weld and the copper side, with AlCu phases distributed between the Al_2_Cu strips.

At the bottom region of the weld shown in Figure 8d, the phase distribution characteristics were similar to those at the shoulder interface. Point 8, located at the deepest part of the weld, was tentatively identified as a Cu-rich phase of Cu + Al_4_Cu_9_. Point 9 was tentatively identified as the AlCu phase, Point 10 was tentatively identified as the Al_2_Cu phase, and Point 11 was tentatively identified as the α-Al + Al-Cu eutectic phase.

Combined with the EDS line scanning results in Figure 8e, it can be seen that under this low heat input welding process (P1S1), the diffusion width of Cu elements into the aluminum weld was approximately 12 μm, with a steep concentration gradient at the interface. The limited elemental diffusion led to significant regional differences in solidification behavior: the central region of the weld was dominated by α-Al and low-Cu Al-Cu phases, while the interfacial region was enriched with Cu-rich phases.

When the laser power was increased to 3.8 kW and the welding speed was reduced to 1.2 m/min, the morphology and composition of the lap joint were as shown in Figure 9. Figure 9a presents the macro-structure of the weld, while the EDS analysis results of the same regions under the previous condition are shown in Figure 9b–d. The chemical compositions at the marked points (yellow “+”) in Figure 9b–d are listed in Table 5. In the middle region of the weld (Figure 9b), chemical composition analysis showed that Point 1 was tentatively identified as Al-Cu eutectic + Al_4_Cu_9_, and Point 2 was tentatively identified as α-Al + Al_2_Cu. With the significant increase in welding heat input, on the one hand, the high-temperature residence time of the weld is prolonged, promoting the diffusion of Cu elements from the base metal over a longer distance toward the weld center. On the other hand, the cooling rate of the weld is reduced, providing kinetic conditions for the uniform distribution of Cu elements in the weld center and the nucleation of Cu-containing phases. Therefore, compared with the α-Al-dominated microstructure in Figure 8b, a mixed structure containing Cu-rich phases formed in this region, and the degree of elemental mixing was greatly enhanced. Concurrently, the microstructure also changed: the content of the Al_2_Cu phase (IMCs) decreased, and its morphology transformed from a network-like structure to a fine blocky structure, which is beneficial for improving the strength and toughness of the joint.

The microstructure and EDS results at the shoulder interface near the copper side are shown in Figure 9c. A more pronounced transition layer can be observed at the weld cross-section. The chemical composition at Point 3 indicates that the phase is supersaturated α-Cu. Along the transition layer, a relatively thick gray structure is present, and Point 4 suggests that this structure consists of AlCu + Al_4_Cu_9_. A small amount of black blocky structure was distributed along the interface, with a copper atomic percentage of 34.78%, corresponding to the Al_2_Cu phase (IMCs). The structures at Points 6 and 7 were similar to those in the middle region of the weld, identified as α-Al + Al_2_Cu and Al-Cu eutectic + Al_4_Cu_9_, respectively. The shoulder interface was located in the transition zone between the weld and the Cu base metal. Under the current process, the relatively high heat input reduces the temperature gradient at the interface, resulting in better matching between the elemental diffusion rate and the solidification rate. During solidification, phase precipitation follows the rule of “continuous composition transition”, gradually transitioning from the supersaturated phase on the Cu side to the Al-Cu compound phases on the weld side, thereby avoiding the abrupt precipitation of Cu-rich phases.

As shown in Figure 9d, at the bottom of the weld, according to the chemical composition analysis results of Points 9 and 10, the white bamboo shoot-like structure consisted of α-Cu + dispersed Al_2_Cu. The combined effect of supersaturated solid solution strengthening and dispersion strengthening significantly improved the strength and hardness of the weld compared with the pure copper base metal. Above the bamboo shoot-like structure, a thick gray-white layer was present, and its composition analysis indicates it as an Al-Cu eutectic. The black blocky structure at Point 12 was the Al_2_Cu phase (IMCs), with a relatively low content. The bottom of the weld was the last region to solidify. Under the current process, the sufficient heat input makes the liquid phase composition in the bottom region more uniform, satisfying the nucleation conditions for the Al-Cu eutectic phase (the eutectic phase requires the liquid composition to approach the eutectic point). Meanwhile, the cooling rate at the bottom interface is relatively moderate, allowing the Al_2_Cu phase to nucleate alternately and disperse uniformly.

The EDS line scanning results in Figure 9e show that the diffusion width of Cu elements into the weld increased to approximately 22 μm, and the interfacial concentration gradient was significantly reduced compared with P1S1. The smoother elemental transition confirms the improved uniformity of elemental distribution within the weld under moderate heat input, which is consistent with the microstructural observation of more thorough molten pool mixing.

When the laser power was increased to 4.0 kW and the welding speed was reduced to 0.9 m/min, the morphology and composition of the Al/Cu laser welded joint are shown in Figure 10. Under these parameters, the welding heat input was the highest. As can be seen from the weld macro-structure in Figure 10a, the right end of the weld exhibited a typical post-weld cracking morphology, while the left end of the weld showed relatively better interfacial formation, allowing analysis of the microstructural evolution of the weld under high heat input conditions. The EDS analysis results are shown in Figure 10b–d, and the chemical compositions at the marked points (yellow “+”) in Figure 10b–d are listed in Table 6. In the middle region of the weld (Figure 10b), the grains were much coarser than those in Figure 8b and Figure 9b. The gray-white structure at Point 1 (mainly composed of hard and brittle CuAl_2_) was arranged in a herringbone-like parallel pattern with an IMC layer thickness exceeding 10 μm and accounting for more than 50% of the region, which can easily lead to a sharp decrease in joint toughness. Figure 10c shows the shoulder interfacial region of the weld, where cracks propagating along the IMC clusters at the Al-Cu interface can be observed. The heat accumulation during welding of this sample increased, and the mixed IMCs at Points 3 and 4 (α-Cu + CuAl_2_) exhibited a large difference in thermal expansion coefficients, resulting in significant welding residual stress. The cracks propagated in multiple directions within the mixed IMCs, with several branches terminating at the interface with the α-Cu + Al_4_Cu_9_ phase, indicating that the mixed IMCs on the interface side are sensitive to cracking and that the Al_4_Cu_9_ phase can suppress crack propagation to some extent. The microstructure at the bottom interface of the weld in Figure 10d was similar to that in Figure 10c, with the mixed IMCs at the interface being even coarser. From the EDS line scanning results in Figure 10e, for the P3S3 sample with excessive heat input, the Cu diffusion width further increased to approximately 36 μm, indicating severe elemental interdiffusion caused by prolonged high-temperature residence time, which provides sufficient kinetic conditions for the coarsening of brittle IMCs.

### 3.3. Joint Shear Strength

**Figure 11:**
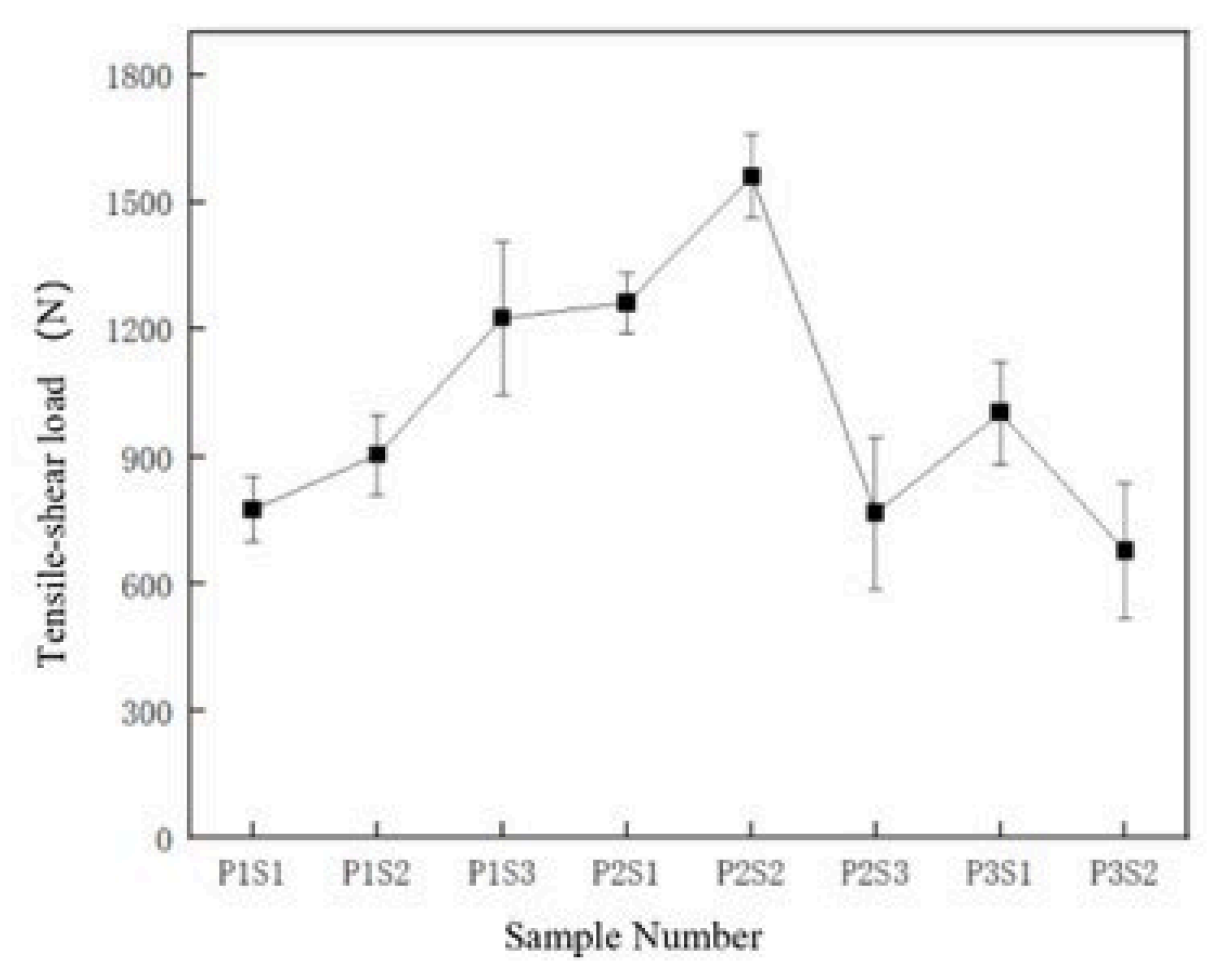
Tensile-shear load of Al/Cu joints under different welding parameters (error bars represent standard deviation, *n* = 3).

As shown in Figure 11, the tensile-shear load results of the various Al/Cu laser welded joints under different parameters exhibited significant regular fluctuations in mechanical properties with varying process parameters. It should be noted that the P3S3 sample with the highest heat input exhibited penetrating transverse cracks in the weld seam due to severe overheating, and could not be machined into complete and valid tensile specimens. Therefore, P3S3 was not included in the tensile-shear performance statistics in Figure 11. For fracture analysis of the high heat input group, the P3S2 sample (4.0 kW + 1.2 m/min) with intact weld formation was selected as the representative, whose heat input (200 kJ/m) remained at a high level and could effectively reflect the interfacial fracture characteristics under high heat input conditions. At a laser power of 3.6 kW, as the welding speed decreased from 1.5 m/min to 0.9 m/min, the joint tensile-shear load gradually increased from 776 N (P1S1) to 1226 N (P1S3). When the laser power was increased to 3.8 kW, the tensile-shear load first increased and then decreased with decreasing welding speed, reaching a peak value of 1561 N at 1.2 m/min (P2S2). Correspondingly, the optimal joint achieved a nominal shear strength of approximately 52.0 MPa, corresponding to 68.5% of the tensile strength of the AA1060 base metal. When the laser power was further increased to 4.0 kW, the tensile-shear load exhibited an overall declining trend due to excessive welding heat input. The macroscopic mechanical properties of the Al/Cu laser welded joints are strongly influenced by the heat input. An appropriate heat input (e.g., 3.8 kW + 1.2 m/min) can improve the joint mechanical properties by optimizing elemental mixing and controlling phase composition and distribution. In contrast, either insufficient heat input (inadequate elemental mixing) or excessive heat input (excessive IMC growth/joint failure) leads to a decrease in tensile-shear load.

## 4. Discussion

This study systematically investigated the effects of laser power and welding speed on the macromorphology, elemental diffusion, microstructure, and mechanical properties of laser welded joints of dissimilar Al/Cu metals. Based on the experimental results, an in-depth comprehensive analysis was conducted from the perspectives of molten pool flow behavior and elemental mixing mechanism, molten pool solidification behavior, and IMC formation mechanism, as well as fracture behavior and mechanism analysis, to elucidate the intrinsic relationship between process parameters and joint performance.

### 4.1. Molten Pool Flow Behavior and Elemental Mixing Mechanism

During laser welding, the flow behavior of the molten pool directly affects the degree and distribution of Al and Cu mixing. Under low heat input conditions (e.g., P1S1: 3.6 kW—1.5 m/min), the molten pool exhibited a large temperature gradient and a short lifetime, resulting in limited convective mixing between the Al and Cu molten pools. The melting amount on the copper side was small, and elemental mixing mainly occurred in the region near the interface (Figure 8). Under such conditions, the Marangoni convection in the molten pool is weak [17], the migration of Cu elements into the Al-side molten pool is limited, and the penetration of Al elements into the Cu-side molten pool is insufficient, leading to a narrow interfacial bonding zone.

With a moderate increase in heat input (P2S2: 3.8 kW—1.2 m/min), the high-temperature residence time of the molten pool is prolonged, the temperature gradient decreases, and Marangoni convection is enhanced, significantly promoting the mixing of Al and Cu molten pools and the homogenization of elements (Figure 9). A relatively uniform Al-Cu mixed region appears in the middle of the molten pool, and a continuous transition layer forms at the interface, which helps alleviate composition abrupt changes and stress concentration. At this stage, the Al content in the Cu-side molten pool increases, and the Cu content in the Al-side molten pool rises, achieving good metallurgical fusion on both sides of the interface.

When the heat input is excessively high (P3S3: 4.0 kW—0.9 m/min), the molten pool becomes overheated, aluminum vaporization intensifies, and the vapor recoil pressure increases, leading to molten pool instability and a tendency for porosity and spatter formation (Figure 10). Moreover, although the excessively long liquid residence time promotes elemental diffusion, it also exacerbates the excessive mixing of Al and Cu, promoting the continuous and coarse growth of brittle IMCs (e.g., Al_2_Cu). Under the combined action of thermal stress and high brittleness of the continuous IMC layer, interfacial microcracks and even solidification cracks are easily initiated [18,19].

### 4.2. Molten Pool Solidification Behavior and IMC Formation Mechanism

**Figure 12:**
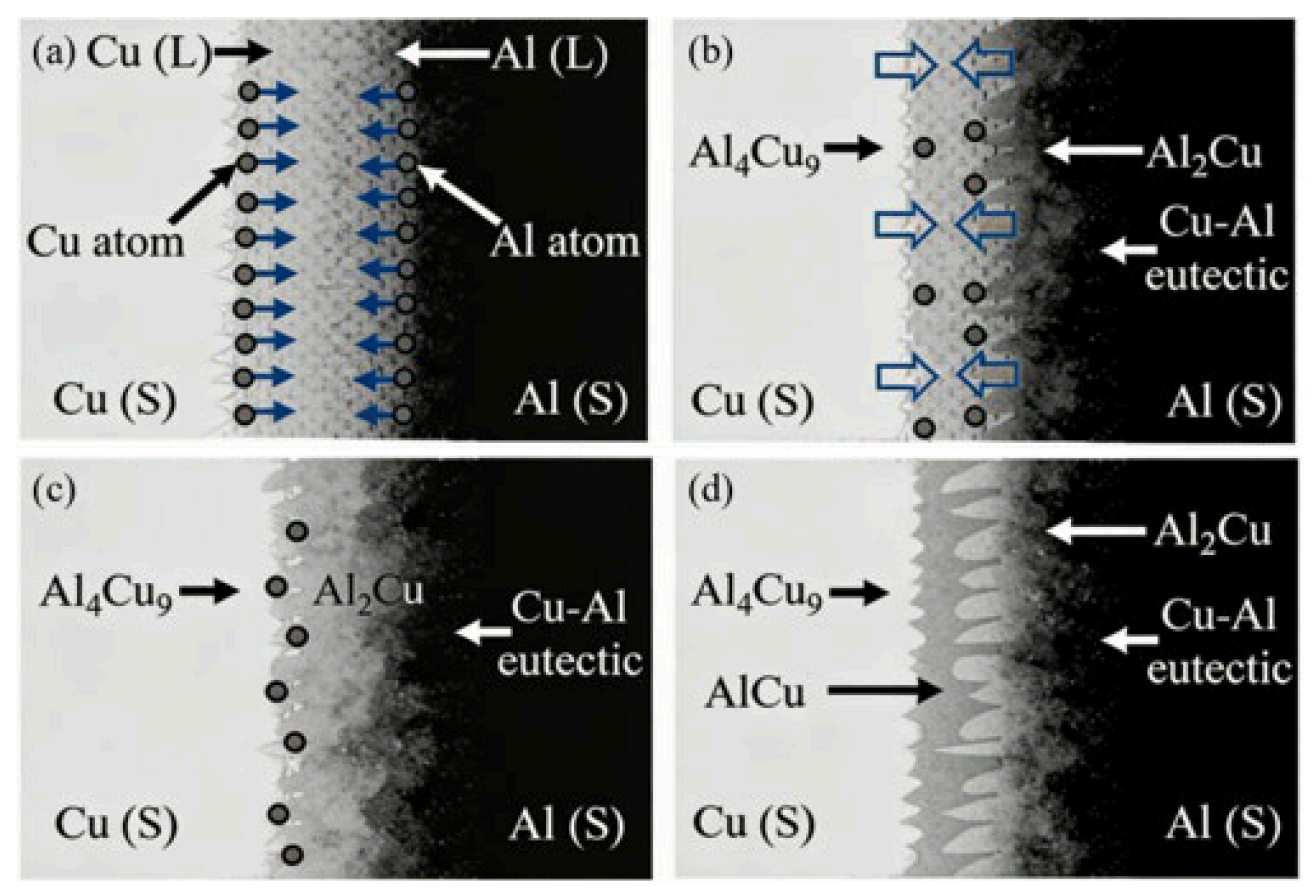
The schematic diagram of intermetallic compound (IMC) formation in the Al-Cu joint. (**a**) The matrix melts and mixes with each other. (**b**) Al2Cu and Al4Cu9 form. (**c**) Al2Cu and Al4Cu9 continue to grow. (**d**) AlCu forms. The blue arrow represents the direction of atomic motion and compound growth.

The type, morphology, and distribution of IMCs at the Al/Cu interface mainly depend on the elemental distribution and cooling conditions during solidification of the molten pool. During laser welding, both the aluminum and copper base metals melt, and the liquid Al and liquid Cu mix through convection and diffusion in the molten pool. As the temperature of the molten pool decreases, when the Cu concentration in the liquid metal reaches a certain value, Al-Cu intermetallic compounds (IMCs) precipitate directly from the liquid phase. According to the Al-Cu binary phase diagram, under non-equilibrium solidification conditions, phases such as Al_2_Cu, AlCu, and Al_4_Cu_9_ may form sequentially. The XRD and EDS results of this study (Figure 7, Table 4, Table 5 and Table 6) show that Al_2_Cu, AlCu, and Al_4_Cu_9_ phases were detected in all joints, and this sequential precipitation and transformation process is schematically summarized by the micro-zone formation mechanism illustrated in Figure 12. Referring to the study by Li et al. [20], the distribution of IMCs is closely related to the distribution of Cu-rich zones in the molten pool: Al_4_Cu_9_ preferentially forms in Cu-rich zones, with the Cu content gradually decreasing outward due to diffusion; in Al-rich zones, Al_2_Cu forms first and is eventually replaced by Al_2_Cu and Al-Cu eutectics.

A schematic diagram of IMC formation in the micro-zone of the Al–Cu interface is shown in Figure 12, which was constructed based on the SEM/EDS experimental observations of this study, combined with the Al-Cu binary phase diagram and IMC growth mechanisms widely verified by multiple independent studies [21,22]. Figure 12a shows that the Al and Cu base metals melt and mix under laser action, which was directly observed from the cross-sectional morphology of the weld. Figure 12b indicates that as the temperature decreases, Al_2_Cu and Al_4_Cu_9_ phases first form in the Al-rich and Cu-rich zones, respectively, which was confirmed by the layered phase distribution at the interface shown in Figure 8, Figure 9 and Figure 10. Figure 12c shows that the IMCs continue to grow as solidification proceeds; in Figure 12d, Al_2_Cu and Al_4_Cu_9_ react to form the AlCu phase. The latter two steps are deduced by combining the solidification law of the Al-Cu binary phase diagram and the conclusions of previous classical studies, which is also consistent with the multi-phase coexistence characteristics detected by XRD in this work.

Under low heat input (P1S1), the molten pool cools rapidly, elemental mixing is insufficient, and the IMCs mainly consist of discrete network-like or blocky Al_2_Cu with uneven distribution, resulting in weak interfacial bonding (Figure 8). A moderately increased heat input (P2S2) reduces the cooling rate, prolongs the molten pool lifetime, promotes uniform elemental mixing, and transforms the IMC morphology from network-like to fine blocky. A continuous and moderately thick Al-Cu eutectic and supersaturated solid solution form at the interface (Figure 9), effectively enhancing interfacial toughness. In contrast, excessively high heat input (P3S3) leads to molten pool overheating, substantial dissolution of Cu into the Al liquid, and coarsening of IMCs upon solidification. The Al_2_Cu phase exhibits coarse dendrites or a continuous layered distribution (Figure 10). Moreover, due to the large difference in thermal expansion coefficients between Al and Cu, significant residual stress develops at the interface, promoting the initiation and propagation of microcracks along IMC clusters [21].

### 4.3. Fracture Behavior and Correlation with Performance

**Figure 13:**
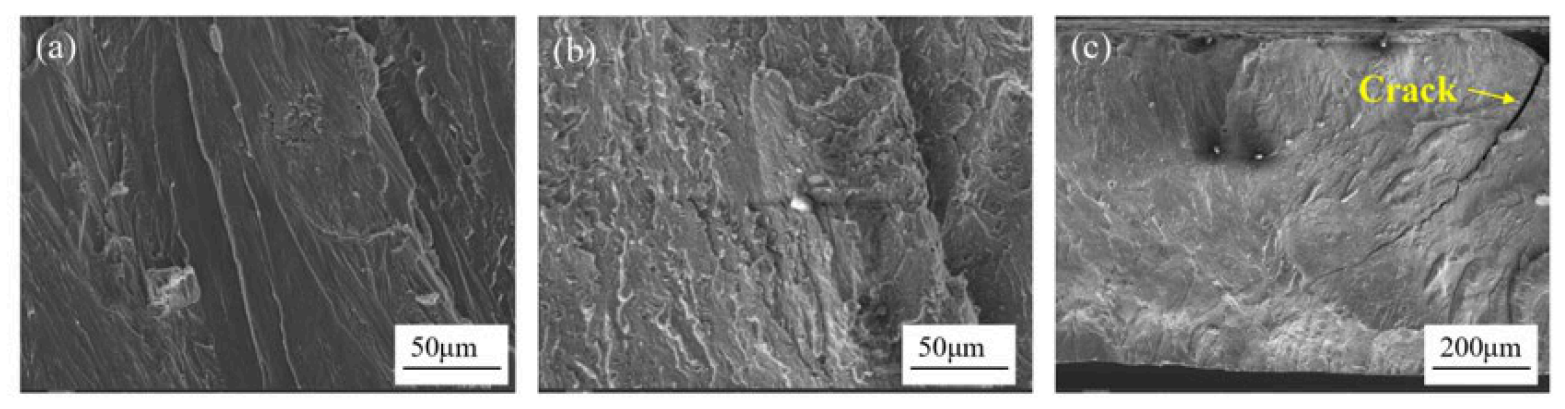
Fracture morphologies of the joints: (**a**) SEM image of P1S1 fracture; (**b**) SEM image of the P2S2 fracture; (**c**) SEM image of the P3S2 fracture.

The shear fracture path of the joint is closely related to the IMC distribution, the penetration depth of the Al-side molten pool, and the interfacial stress state. As shown in Figure 13a, the fracture surface of the low heat input parameter P1S1 (3.6 kW—1.5 m/min) was relatively flat overall, exhibiting typical brittle fracture characteristics. At higher magnification, obvious cleavage steps and river patterns could be observed, and the fracture path mainly propagated along the Al_2_Cu/Al interface, indicating that the brittle IMC layer serves as a preferential channel for crack initiation and propagation. Under this parameter, the interfacial bonding zone is narrow and the IMCs are discretely distributed, making it difficult to effectively hinder crack propagation, thus resulting in a low shear strength (776 N).

The fracture morphology of the optimal parameter P2S2 (3.8 kW—1.2 m/min) is shown in Figure 13b, exhibiting typical ductile-brittle mixed fracture characteristics. A large number of fine dimples (indicated by arrows in the figure) were distributed on the fracture surface, along with some cleavage planes. The formation of dimples is related to the continuously distributed Al-Cu eutectic structure and the fine, dispersed IMCs at the interface—these ductile phases undergo plastic deformation during fracture, absorbing fracture energy and effectively retarding crack propagation. Furthermore, the thorough mixing of the Al and Cu molten pools increases the interfacial bonding strength and prolongs the crack propagation path.

The fracture surface of the high heat input parameter P3S2 (4.0 kW—1.2 m/min) is shown in Figure 13c, again exhibiting brittle fracture characteristics, but with a fracture mode different from that of P1S1. Coarse cleavage planes of the Al_2_Cu phase and intergranular cracks can be observed on the fracture surface, with secondary cracks present in local regions (indicated by arrows in the figure). This is because excessive heat input leads to the overgrowth of IMCs and severe interfacial embrittlement. Meanwhile, residual stress caused by the difference in thermal expansion coefficients between Al and Cu promotes the pre-initiation of microcracks within the IMC clusters or at the interface, leading to rapid crack propagation under shear loading [22], resulting in joint failure at a relatively low load (shear strength decreasing to 678 N).

The fracture morphology analysis indicates that the distribution characteristics of IMCs and the formation of ductile phases at the interface are key factors determining the fracture mode and joint strength. Refining the IMCs and achieving a continuous distribution of the eutectic structure through heat-input regulation can promote a transition of the fracture mode from brittle fracture to ductile-brittle mixed fracture, thereby significantly improving the mechanical properties of the joint.

To further clarify the performance level of the joints in this study, a quantitative comparison with published Al/Cu welding results was conducted. For Al/Cu laser lap welded joints without filler metals or interlayers, the shear strength of thin-sheet joints (≤1 mm) generally ranges from 40 to 55 MPa, while the 52.0 MPa obtained in this study for 2 mm medium-thickness plates reaches the advanced level of similar processes. Compared with processes using interlayers or filler metals (e.g., the 175.5 MPa shear strength of plasma arc welding-brazing with Zn-Al filler reported by Peng et al. [14]), it should be noted that the base aluminum alloy used in Ref. [14] was LF6 aluminum with a tensile strength of approximately 300 MPa, which was significantly higher than the AA1060 pure aluminum (76 MPa) adopted in this work. From the perspective of the joint strength coefficient (the ratio of joint shear strength to base metal tensile strength), the joint in this study reached 68.5% of the base metal strength, which was higher than the approximately 58% in Ref. [14], and it avoids the additional material cost and process complexity introduced by filler metals, showing better economy for automated mass production.

The mechanical properties of the various phases at the Al/Cu interface differed considerably. The microhardness of α-Al was approximately HV 20–30, while that of α-Cu was about HV 60–100. In contrast, the brittle IMC phases—such as Al_2_Cu, AlCu, and Al_4_Cu_9_—exhibited hardness values far exceeding those of the base metals, reaching up to HV 200–400, HV 400–600, and HV 500–700, respectively [23,24]. This significant hardness mismatch renders the continuous IMC layer a preferential path for crack initiation and propagation, which well explains the brittle fracture characteristics observed in joints produced under both low and excessive heat input.

In terms of IMC layer thickness, the optimal thickness of 3–5 μm obtained in this study is consistent with the widely recognized performance threshold in Al/Cu welding research [25,26]. When the IMC layer thickness is controlled below 5 μm, the joint can achieve a good balance between bonding strength and toughness. In contrast to studies on thin sheets (≤1 mm), the molten pool in the present work for 2 mm medium-thickness plates exhibited a substantially longer high-temperature residence time and a steeper thermal gradient, which significantly altered the growth kinetics of the IMCs. Consequently, restricting the IMC layer thickness below this widely recognized threshold (5 μm) is considerably more challenging for medium-thickness plates. Nevertheless, the process window identified in this study (heat input of 144–190 kJ/m) successfully achieved this thickness control, thereby validating its practical applicability and reliability for joining medium-thickness Al/Cu plates.

In summary, this study establishes a complete logical chain of “process parameters → heat input → molten pool behavior and solidification conditions → IMC characteristics (type, morphology, distribution, thickness) → interfacial stress distribution → crack initiation and propagation → fracture mode → joint mechanical properties”. The coupling of laser power and welding speed determines the heat input, which in turn controls the peak temperature of the molten pool, the high-temperature residence time, and the cooling rate. Under low heat input (e.g., P1S1), the molten pool has a short lifetime and a fast cooling rate. The IMCs mainly consist of discrete network-like or blocky Al_2_Cu with an uneven distribution, resulting in weak interfacial bonding. Cracks tend to propagate rapidly along the brittle IMC layer, exhibiting typical brittle fracture characteristics and low shear strength. Under a moderate heat input (e.g., P2S2), the Al and Cu molten pools are thoroughly mixed, and elemental diffusion and alloying reactions proceed smoothly. A continuous Al-Cu eutectic layer and fine, dispersed Al_2_Cu, AlCu, and Al_4_Cu_9_ phases form at the interface. The IMC layer thickness is controlled within 3–5 μm and is alternately distributed with the ductile eutectic structure, effectively alleviating stress concentration caused by the continuous distribution of brittle phases. Meanwhile, the thorough mixing of the Al and Cu molten pools increases interfacial bonding strength and prolongs the crack propagation path. During fracture, the eutectic phase undergoes plastic deformation and absorbs fracture energy, causing the fracture mode to transition from brittle fracture to ductile-brittle mixed fracture, achieving the peak shear strength of 1561 N. Under excessive heat input (e.g., P3S3), the molten pool becomes overheated and has an excessively long lifetime. A large amount of Cu dissolves into the Al liquid, forming coarse and continuous Al_2_Cu dendrites upon solidification. The IMC layer thickness exceeds 10 μm, and the brittleness increases significantly. Moreover, the large difference in thermal expansion coefficients between Al and Cu generates highly concentrated residual stress at the interface, leading to the initiation of microcracks even during cooling. Under shear loading, cracks rapidly propagate along the IMC layer, exhibiting intergranular brittle fracture characteristics and a significant drop in shear strength. These results indicate that by adjusting the laser power and welding speed, the heat input can be effectively controlled, thereby optimizing the morphology and distribution of IMCs, improving the interfacial stress state, and achieving a transition in fracture mode from brittle to ductile-brittle mixed fracture, ultimately obtaining Al/Cu laser welded joints with high strength and toughness. This systematic understanding provides a theoretical basis for the rational design and performance prediction of dissimilar Al/Cu joining processes.

## 5. Conclusions

In this paper, the effects of laser power and welding speed on the weld formation, interfacial microstructure, and mechanical properties of laser-welded joints of AA1060 aluminum and T2 copper were systematically investigated. The following conclusions are drawn:

(1) Heat input is a key parameter determining weld formation and interfacial microstructure characteristics. Under low heat input, the penetration depth on the aluminum side is shallow, the interfacial reaction on the copper side is insufficient, and the IMCs mainly consist of discrete network-like Al_2_Cu with uneven distribution. Under a moderate heat input, the molten pool on the aluminum side expands sufficiently, and a continuous Al-Cu eutectic layer together with fine, dispersed Al_2_Cu, AlCu, and Al_4_Cu_9_ phases forms on the copper side. Under excessive heat input, the interfacial IMCs coarsen into a continuous layered structure, the proportion of CuAl_2_ dendrites exceeds 50%, and crack initiation occurs, inducing post-weld cracking.
(2) The joint shear strength first increases and then decreases with increasing heat input, reaching a peak value of 1561 N under the moderate heat input parameters of 3.8 kW—1.2 m/min. Under these parameters, the interfacial microstructure is characterized by an alternating distribution of fine, dispersed IMCs and a continuous Al-Cu eutectic layer. The fracture mode transforms from brittle fracture under low heat input to ductile-brittle mixed fracture, with the eutectic phase absorbing fracture energy through plastic deformation, thereby effectively improving joint strength and toughness.
(3) A suitable process window for the laser welding of 2 mm-thick AA1060 aluminum to T2 copper was identified: laser power of 3.6–3.8 kW, welding speed of 1.2–1.5 m/min (heat input of 144–190 kJ/m). Within this window, controllable optimization of the morphology and distribution of interfacial IMCs can be achieved by adjusting the heat input, resulting in high-strength and high-toughness joints. The findings provide a theoretical basis for the process design of the laser welding of dissimilar Al/Cu medium-thick plates.

Future research will be extended in the following directions: (1) laser welding of medium-thickness Al/Cu plates assisted by filler wires or intermediate coatings, to further achieve the precise regulation of interfacial intermetallic compounds and improve the comprehensive performance of joints; (2) welding behavior of recycled Al/Cu materials to clarify the influence mechanism of impurity elements on interfacial metallurgical reaction and joint properties; (3) multi-scale mechanical characterization of the joint interface, including microhardness, nanoindentation, and fatigue performance tests, to quantitatively describe the gradient distribution of interfacial mechanical properties and improve the multi-dimensional performance evaluation system; (4) combination of numerical simulation and experimental research to quantitatively analyze the molten pool flow behavior and IMC growth kinetics, and realize accurate prediction of the welding process and joint properties.

## Figures and Tables

**Table 1:** Chemical composition of AA1060 aluminum (wt.%).

Al	Mn	Si	Mg	Zn	Ti
99.6	0.03	0.25	0.03	0.06	0.03

**Table 2:** Chemical composition of T2 copper (wt.%).

Cu	Bi	Zn	S	As	Fe
99.7	0.001	0.2	0.002	0.002	0.005

**Table 3:** Laser welding process parameters and sample designations.

Process No.	Laser Power (kW)	Welding Speed (m/min)	Heat Input (kJ/m)
P1S1	3.6	1.5	144
P1S2	3.6	1.2	180
P1S3	3.6	0.9	240
P2S1	3.8	1.5	152
P2S2	3.8	1.2	190
P2S3	3.8	0.9	253
P3S1	4.0	1.5	160
P3S2	4.0	1.2	200
P3S3	4.0	0.9	266

**Table 4:** Chemical compositions of the marked points in Figure 8b–d.

Test Point	Al (at. %)	Cu (at. %)	Possible Phase
1	95.39	4.61	α-Al
2	64.58	35.42	α-Al + Al_2_Cu
3	24.83	76.17	Cu + Al_4_Cu_9_
4	44.01	55.99	AlCu
5	69.14	30.86	Al_2_Cu
6	95.97	4.03	α-Al + Al eutectic
7	72.40	27.60	α-Al + Al_2_Cu
8	21.55	78.45	Cu + Al_4_Cu_9_
9	46.13	53.87	AlCu
10	70.70	29.30	Al_2_Cu
11	94.03	5.97	α-Al + Al eutectic
12	67.66	32.34	α-Al + Al_2_Cu

**Table 5:** Chemical compositions of the marked points in Figure 9b–d.

Test Point	Al (at. %)	Cu (at. %)	Possible Phase
1	34.96	65.04	AlCu + Al_4_Cu_9_
2	65.62	34.38	α-Al + Al_2_Cu
3	17.72	82.28	α-Cu(Al)
4	34.90	65.10	AlCu + Al_4_Cu_9_
5	65.22	34.78	Al_2_Cu
6	74.54	25.46	α-Al + Al_2_Cu
7	32.98	67.02	AlCu + Al_4_Cu_9_
8	28.46	71.54	Al_4_Cu_9_
9	18.52	81.48	α-Cu + Al_2_Cu
10	21.58	78.42	α-Cu + Al_2_Cu
11	33.39	66.61	Al-Cu eutectic
12	76.41	23.59	α-Al + Al_2_Cu

**Table 6:** Chemical compositions of the marked points in Figure 10b–d.

Test Point	Al (at. %)	Cu (at. %)	Possible Phase
1	20.24	79.76	α-Cu + CuAl_2_
2	38.33	61.67	α-Cu + Al_4_Cu_9_
3	16.03	83.97	α-Cu + CuAl_2_
4	20.53	79.47	α-Cu + CuAl_2_
5	20.44	79.56	α-Cu + CuAl_2_
6	35.28	64.72	α-Cu + Al_4_Cu_9_
7	16.58	83.42	α-Cu + CuAl_2_
8	15.87	84.13	α-Cu + CuAl_2_
9	17.50	82.50	α-Cu + CuAl_2_
10	18.00	82.00	α-Cu + CuAl_2_
11	42.23	57.77	α-Cu + Al_4_Cu_9_
12	39.86	60.14	α-Cu + Al_4_Cu_9_

## Data Availability

The original contributions presented in this study are included in the article. Further inquiries can be directed to the corresponding author.
